# Peripheral dentinogenic ghost cell tumor of the ethmoid sinus: A case report

**DOI:** 10.1097/MD.0000000000018896

**Published:** 2020-01-17

**Authors:** Guo Liu, Jin-nan Li, Feng Liu

**Affiliations:** a Department of Otolaryngology-Head and Neck Surgery; b Department of Pathology, West China Hospital of Sichuan University, Chengdu, China.

**Keywords:** child, dentinogenic ghost cell tumor, odontogenic tumors, paranasal sinus

## Abstract

**Rationale:**

The dentinogenic ghost cell tumor (DGCT), a locally invasive benign neoplasm, is one of the rarest odontogenic tumors, usually developing in the maxilla or mandible. It can be classified into 2 types: intraosseous (central) and extraosseous (peripheral). Here, we describe the first case of a peripheral DGCT located in the ethmoid sinus.

**Patient concerns:**

An 8-year-old boy presented to our department with a longer than 7-month history of nasal obstruction, purulent secretion, and reduction in sense of smell in the right nasal cavity.

**Diagnosis:**

The patient was diagnosed with peripheral DGCT of the ethmoid sinus based on computed tomography scan and pathology.

**Interventions:**

Functional endoscopic sinus surgery was performed.

**Outcomes:**

With 2 years of follow-up, there was no evidence of recurrence.

**Lessons:**

Peripheral DGCT can occur in the paranasal sinus and the need to consider this entity as a possible diagnosis by the clinicians.

## 1 Introduction

The calcifying cystic odontogenic tumor (CCOT), a term formally used since it was named by the World Health Organization in 2005,^[1]^ was first described as a distinct clinicopathologic entity in 1962 by Gorlin et al.^[2]^ The dentinogenic ghost cell tumor (DGCT) is the solid variant of CCOT and is characterized by ameloblastoma-like epithelial cells associated with ghost cells and dysplastic dentin.^[1]^

The DGCT is one of the rarest odontogenic tumors, regarded as a locally invasive benign neoplasm. It may present as an intraosseous (central) or extraosseous (peripheral) process.^[3]^ The intraosseous DGCT is reported to be locally invasive, and patient age ranges from 12 to 75 years,^[4]^ whereas the extraosseous type is extremely rare and less aggressive, with an age range of 7 to 92 years.^[5]^ No matter what type, the DGCT usually occurs in the maxilla and mandible.^[4,5]^

Here, we present a case of peripheral DGCT located in the right ethmoid sinus in an 8-year-old child. To our knowledge, this is the first report of a primary DGCT in the paranasal sinus in the available English literature. The patient's parents have provided informed consent for publication and this report was also approved by the Ethics committee of the West China Hospital, Sichuan University.

## 2 Case report

An 8-year-old boy presented to the pediatric department with a longer than 7-month history of nasal obstruction, purulent secretion, and reduction in sense of smell in the right nasal cavity. After drug treatment was ineffective, he was advised to visit our Ear, Nose, and Throat department. The patient had no history of nasal foreign bodies, fever, headache, facial pain, or bloody secretion. No lymphadenopathy was noted.

**Figure 1:**
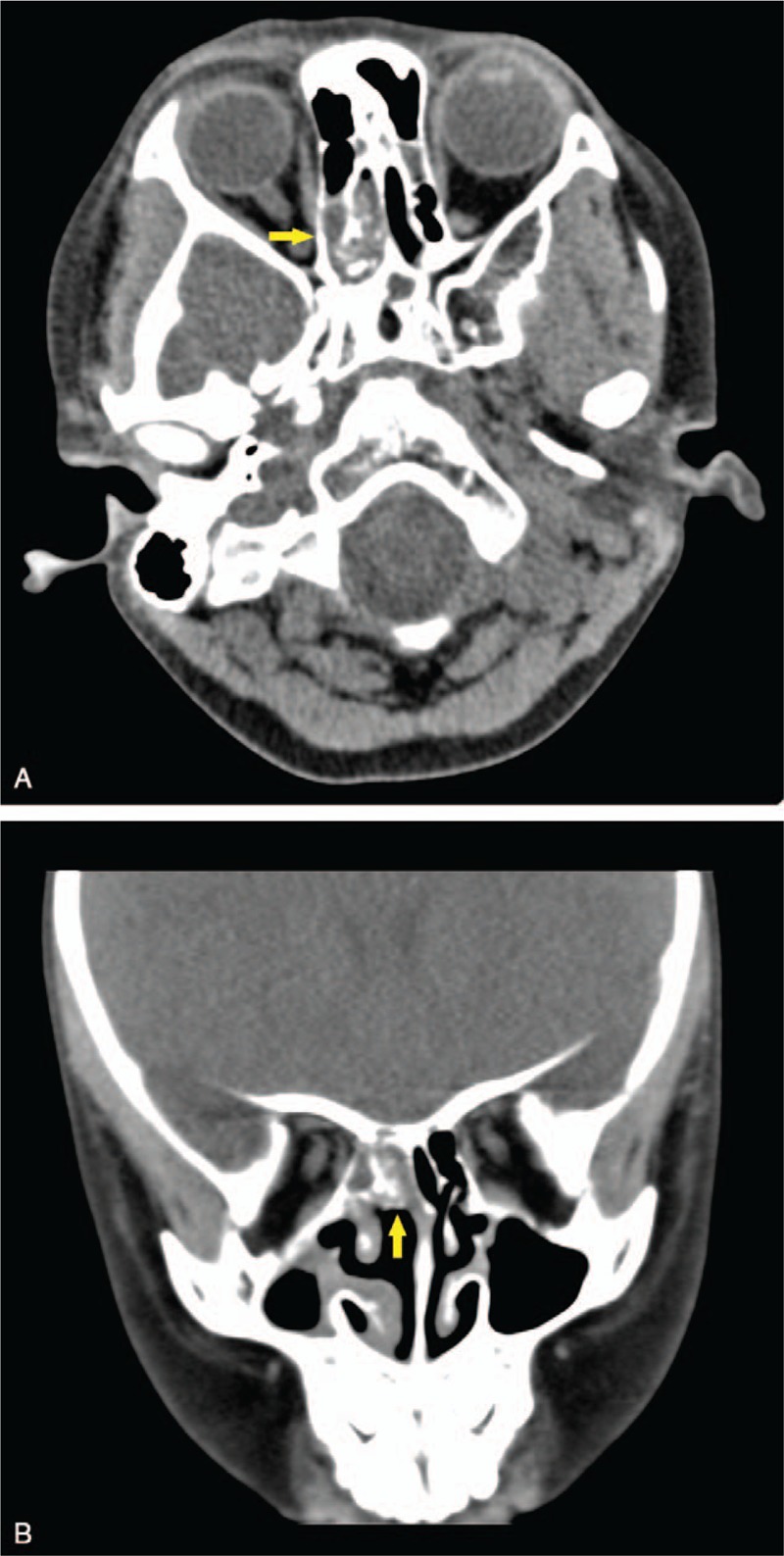
Computed tomography revealed soft tissue density mixed with multiple discrete hyperdense calcification in the right posterior ethmoid sinus (yellow arrow).

Considering that the patient might not be able to cooperate with nasal endoscopy, a computed tomography (CT) examination was performed and revealed soft tissue density mixed with multiple discrete hyperdense calcification in the right posterior ethmoid sinus, without bony destruction (Fig. 1). Based on CT features and symptoms, our first diagnosis was fungal rhinosinusitis. Differential diagnosis included benign masses of the paranasal sinus.

**Figure 2:**
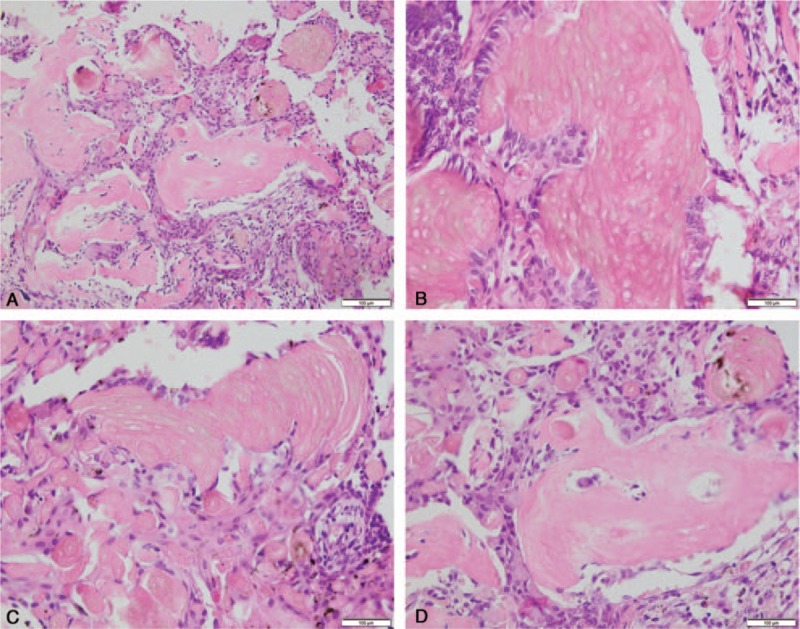
Histopathological findings. (A) Photomicrograph of the lesion showing 1 or 2 islands of odontogenic epithelium with eosinophilic material resembling dentin and ghost cells (H&E, ×40); (B) areas of numerous fused ghost cells with proliferative epithelium (H&E ×100); (C) 1 ameloblastoma-like island of odontogenic epithelium with clusters of ghost cells (H&E ×100); (D) variable amounts of dentinoid material (H&E ×100). H&E = hematoxylin and eosin.

We performed endoscopic sinus surgery for the patient under general anesthesia, and found that the lesion was a solid mass of about 1.9 cm × 1.2 cm × 1.1 cm with a smooth surface involving the olfactory area. After complete resection, the specimens were sent for histopathological analysis. The hematoxylin and eosin-stained section showed proliferative surface epithelium without any dysplasia and 1 or 2 islands of odontogenic epithelium with eosinophilic material resembling dentin and numerous ghost cells (Fig. 2). The pathological diagnosis was DGCT. After examining the gums, maxilla, and mandible without lesions, the patient was finally diagnosed as peripheral DGCT of the ethmoid sinus. With 2 years of follow-up, there was no evidence of recurrence.

## 3 Discussion

Peripheral DGCT is extremely rare. According to a systematic review in 2018,^[6]^ 15 cases have been reported. However, other literature suggested that the number of cases was much more than that.^[5]^ After we reviewed the available English literature with the search term of “dentinogenic ghost cell tumor,” 25 cases of peripheral DGCT were finally found, only 1 case of which was a child (Table 1). Here, we report another case of peripheral DGCT originating from the ethmoid sinus in an 8-year-old child. Although there were some reports of DGCT involving the maxillary sinus, they all originated from the maxillary alveolus.^[7,8]^ Therefore, to our knowledge, this report is the first case of a primary DGCT in the paranasal sinus.

**Table 1:**
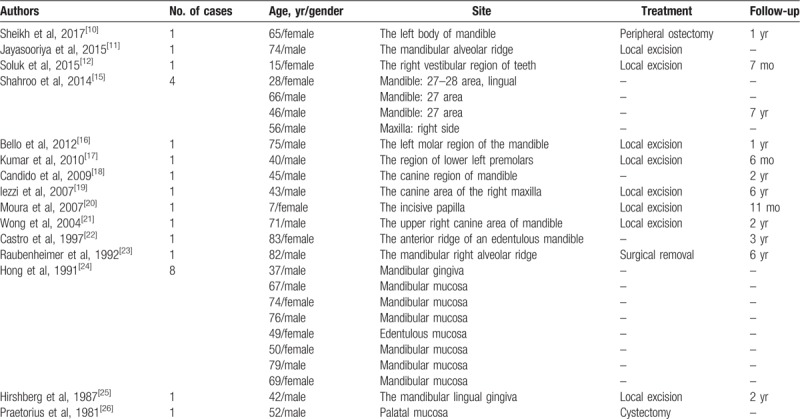
Review of the literatures describing peripheral dentinogenic ghost cell tumor.

DGCT is a locally aggressive neoplasm that usually develops in the maxilla or mandible, accounting for less than 1% of all odontogenic tumors.^[6]^ It is classified into 2 types: intraosseous (central, 83%) and extraosseous (peripheral, 17%).^[3]^

The central DGCTs are usually located centrally in the bone (the maxilla and mandible), mainly occur in the canine to first molar region and exhibit as an asymptomatic swelling, although some may present with slight numbness and pain.^[4,9]^ Most peripheral DGCTs originate from the gingiva in dentate patients and alveolar mucosa in edentulous patients, usually appear as a sessile, sometimes pedunculated, exophytic mass.^[5,10]^ In our case, the lesion might be derived from the mucosa of the nasal or ethmoid sinus, and involved the nasal cavity and olfactory area. Therefore, nasal symptoms similar to the symptoms of chronic sinusitis became the chief complaints. Nasal congestion and reduction in sense of smell caused by mechanical obstruction, and the purulent secretion might be complicated with the maxillary sinusitis. In addition, DGCTs usually appear radiographically as a cystic lesion or soft mass mixed radiolucent–radiopaque lesion depending upon the degree of calcification, which are easily confused with the CT features of fungal ball in nasal sinus. Therefore, it might be misdiagnosed as fungal sinusitis. However, fungal sinusitis in children is very rare, so differential diagnosis should be considered, such as ameloblastoma, ossifying fibroma, and osteodysplasia fibrosa.

Accurate diagnosis depends on pathological findings. Actually, both central and peripheral variants of DGCTs exhibit similar histological features in that the tumors are composed of ameloblastoma-like islands of odontogenic epithelium with clusters of ghost cells and variable amounts of dentinoid material in the surrounding tissue or near the epithelium.^[1,11]^ The ghost cells appear as eosinophilic epithelial cells that have lost their nuclei. The islands of ameloblastoma-like epithelium with ghost cells can distinguish DGCT from ossifying fibroma and osteodysplasia fibrosa but they may be difficult to separate from ameloblastoma. If it happens, dysplastic dentin can provide more information because the dentin and ghost cells are generally not present in ameloblastoma simultaneously.^[12]^ Lack of cystic structure and atypical mitosis can be used to distinguish from CCOT and ghost cell odontogenic carcinoma, respectively.^[12]^

Therapeutically, central DGCTs have an aggressive behavior and a high recurrence so that extensive surgical resection with an adequate safety margin is recommended.^[4]^ Peripheral DGCTs are less aggressive and can be controlled by local complete excision.^[5]^ No recurrences have been reported.^[5,12]^ Regardless of the type, radiotherapy and chemotherapy are not recommended. On rare occasions, the DGCT transforms into ghost cell odontogenic carcinoma.^[13]^ Therefore, a long-term follow-up of no less than 3 years is suggested.^[5]^ There are no significant differences in clinical manifestations, pathology, and treatment between adults and children, according to current reports.^[14]^

In summary, peripheral DGCT can occur in both adults and children without gender predilection. The sinus DGCT may be confused with fungal sinusitis based on the clinical manifestations, which reminds clinicians to consider this entity as a possible diagnosis.

## Author contributions

**Conceptualization:** Guo Liu.

**Data curation:** Guo Liu, Jin-nan Li.

**Methodology:** Feng Liu.

**Resources:** Feng Liu.

**Supervision:** Feng Liu.

**Writing – original draft:** Guo Liu.

**Writing – review and editing:** Feng Liu.
